# The Association Between Serum Immunoglobulin G Titers Against *Porphyromonas gingivalis* and Chronic Kidney Disease in Patients With Rheumatoid Arthritis and Periodontitis: A Retrospective Cohort Study

**DOI:** 10.1002/cre2.70271

**Published:** 2026-01-29

**Authors:** Tetsuo Kobayashi, Satoshi Ito, Noriko Sugita, Akira Murasawa, Hajime Ishikawa, Koichi Tabeta

**Affiliations:** ^1^ Department of Dentistry National Hospital Organization Niigata National Hospital Kashiwazaki Japan; ^2^ Division of Periodontology, Department of Oral Biological Science Niigata University Graduate School of Medical and Dental Sciences Niigata Japan; ^3^ Department of Rheumatology Niigata Rheumatic Center Shibata Japan

**Keywords:** chronic kidney disease, periodontitis, *Porphyromonas gingivalis*, rheumatoid arthritis

## Abstract

**Objectives:**

Chronic kidney disease (CKD) is relatively common in patients with rheumatoid arthritis (RA). Periodontitis and periodontopathic *Porphyromonas gingivalis* are risk factors for CKD. However, the association of serum immunity to *P. gingivalis* and its peptidylarginine deiminase (PPAD), as well as periodontitis severity, with CKD in relation to RA has not been elucidated. The present study evaluated whether or not serum immunoglobulin G (IgG) titers against *P. gingivalis* and PPAD and periodontitis severity are associated with CKD in patients with RA and periodontitis.

**Methods:**

Demographic, comorbidity, rheumatologic, and periodontal data were collected from 127 patients with RA and periodontitis in a retrospective cohort study. CKD was defined as an estimated glomerular filtration rate (eGFR) < 60 mL/min/1.73 m^2^ and/or proteinuria of ≥ 3 months’ duration. Serum IgG titers against *P. gingivalis* and PPAD were determined using an electrochemiluminescence immunoassay.

**Results:**

Twenty patients showed an eGFR < 60 mL/min/1.73 m^2^, while no patients had proteinuria. The 20 CKD patients were significantly older (*p* = 0.002), had higher percentages of former smokers (*p* = 0.01), had more sites with probing depth and clinical attachment level ≥ 4 mm (*p* = 0.03 and *p* = 0.02), and had higher levels of serum creatinine and eGFR (*p* < 0.001 for both) and anti‐*P. gingivalis* IgG titers (*p* = 0.04) than the 107 non‐CKD patients. A significant association was observed between anti‐*P. gingivalis* IgG titers and eGFR (*p* < 0.001 for both) by bivariate and multivariate analyses and between anti‐*P. gingivalis* IgG titers and CKD (*p* < 0.001) using a multiple logistic regression analysis after adjusting for age, gender, smoking status, comorbidity, RA condition, and RA‐related drugs.

**Conclusions:**

These results suggest that serum IgG titers against *P. gingivalis*, but not against PPAD, are associated with CKD in patients with RA and periodontitis.

## 1 Introduction

Periodontitis is a serious stage of chronic inflammatory periodontal disease caused by periodontopathic bacterial infections, accompanied by irreversible inflammation‐mediated destruction of periodontal tissue (Pihlstrom et al. 2005). Accumulating evidence suggests that periodontitis and periodontopathic *Porphyromonas gingivalis* are causal factors for initiating the autoimmune inflammatory response in rheumatoid arthritis (RA) (Bartold and Lopez‐Oliva 2020; Kobayashi and Bartold 2023), characterized by destruction of the joints, leading to functional limitations (Smolen et al. 2016).

One of the proposed mechanisms underlying periodontitis‐ and *P. gingivalis*‐induced RA involves the increased levels of protein citrullination and subsequent production of autoantibodies to cyclic citrullinated peptide (CCP), accelerated by periodontitis‐induced neutrophil extracellular traps (NETs) and endogenous peptidylarginine deiminase (PAD)‐4 as well as by *P. gingivalis* PAD (PPAD) (Bartold and Lopez‐Oliva 2020; Kobayashi and Bartold 2023). Another plausible causal mechanism involves the overexpression of autoantibodies to immunoglobulin G (IgG), known as rheumatoid factor (RF), and those to agalactosyl IgG, IgG to oligosaccharides that lack terminal galactose residues, both of which are associated with RA and periodontitis (Kaneko et al. 2021; Matsui et al. 2006; Thé and Ebersole 1991).

A variety of kidney disorders are relatively common in patients with RA, with the prevalence of chronic kidney disease (CKD) ranging from 15.0% to 25.4%. The potential causes of CKD in these patients are age‐related physiological changes and comorbid disorders, including cardiovascular disease (CVD), hypertension (HT), diabetes mellitus (DM), dyslipidemia, secondary amyloidosis, and RA drug‐related kidney disorders (Hickson et al. 2014; Karie et al. 2008; Saisho et al. 2016; Tokoroyama et al. 2017). An increased risk of infection has been reported in patients with CKD, some of which contribute to hospitalization due to pneumonia and subsequent comorbidities (Dalrymple and Go 2008; James et al. 2009; Xu et al. 2017).

These observations suggest that serum immunity to *P. gingivalis* and PPAD may be associated with CKD in patients with RA and periodontitis, leading to further development of periodontopathic bacterial infection and periodontitis. However, no study has evaluated the association between serum levels of IgG to *P. gingivalis* and PPAD, and CKD in patients with RA and periodontitis. Therefore, the present study aimed to evaluate whether or not serum IgG titers against *P. gingivalis* and PPAD, as well as periodontitis severity, are associated with CKD in patients with RA and periodontitis.

## 2 Materials and Methods

### 2.1 Study Design and Patients

**Figure 1:**
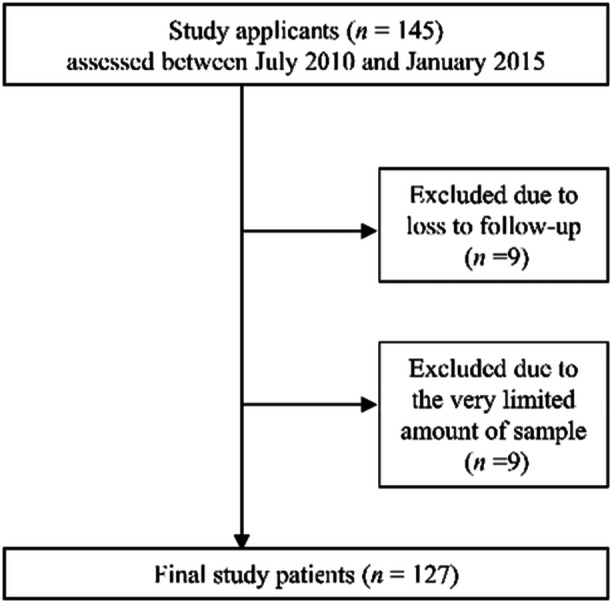
Flowchart of the study patient selection.

This retrospective cohort study was conducted between September 2017 and November 2020, with collection of the demographic, comorbidity, rheumatologic, and periodontal data from the databases of Niigata Rheumatic Center. A total of 145 patients with RA who met the American College of Rheumatology/European League Against Rheumatism 2010 criteria (Aletaha et al. 2010) were selected for the study applicants from 3 previous studies (Kobayashi et al. 2015; Kojima et al. 2016; Okada et al. 2013). Of these applicants, nine patients were excluded because of loss to follow‐up, while another nine were excluded because of the very limited amount of serum samples available. Therefore, the final study population included 127 RA patients (Figure 1).

All patients underwent medical interviews, rheumatologic and periodontal assessments, blood collection, and treatments with conventional synthetic disease‐modifying antirheumatic drugs (csDMARDs), glucocorticoids, nonsteroidal anti‐inflammatory drugs (NSAIDs), or biological DMARDs (bDMARDs) at Niigata Rheumatic Center between July 2010 and January 2015. The eligibility criteria were having ≥ 20 teeth, the absence of pregnancy, and no history or the absence of any periodontal treatment, including plaque control, supragingival and subgingival scaling, root planing, and mouth rinse usage, and antibiotic medication within the previous 3 months.

The present study was approved by the Ethics Committee of Niigata University (Approval Number 2017‐0114) and Niigata Rheumatic Center (Approval Number 2017‐009) and was conducted in accordance with the ethical principles of the Declaration of Helsinki. All patients provided their signed informed consent to participate in the present study.

### 2.2 Demographic and Comorbidity Data

The demographic data included the age, gender, and smoking status (never‐, former‐, or current‐smoker), while the comorbidity data were collected according to the following definitions: CVD was defined as having a history of treatment for myocardial infarction, stroke, or transient ischemic attack (Solomon et al. 2010); HT was defined as having ≥ 2 resting blood pressure measurements of ≥ 140 mmHg systolic and/or ≥ 90 mmHg diastolic or having a history of treatment with antihypertensive drugs (Teramoto et al. 2007); DM was defined as having at least 2 measurements of HbA1c ≥ 6.5% or a fasting plasma glucose level ≥ 126 mg/dL, or having a history of treatment with hypoglycemic drugs (American Diabetes Association 2014; Teramoto et al. 2007); and dyslipidemia was defined as having a low‐density lipoprotein cholesterol level ≥ 140 mg/dL, a high‐density lipoprotein cholesterol level < 40 mg/dL, a triglyceride level ≥ 150 mg/dL, or being treated with specific lipid‐lowering drugs (Teramoto et al. 2007).

### 2.3 Rheumatologic Data

The rheumatologic data obtained by 3 licensed rheumatologists (S.I., A.M., H.I.) were collected from the databases, which included the disease duration, disease activity score in 28 joints using C‐reactive protein (DAS28‐CRP) (Inoue et al. 2007), tender joint count (TJC), swollen joint count (SJC), and patient global assessment (Pt‐GA), as well as RA‐related drugs, which included csDMARDs (e.g. methotrexate, sulfasalazine, bucillamine, tacrolimus, iguratimod, and mizoribine), corticosteroids, NSAIDs, and bDMARDs, which included tumor necrosis factor (TNF) inhibitors (i.e. infliximab [IFX], etanercept [ETN], adalimumab [ADA], golimumab [GLM], certolizumab pegol [CZP], and abatacept [ABT]) and interleukin‐6 receptor (IL‐6R) inhibitors (i.e. tocilizumab [TCZ]).

### 2.4 Periodontal Data

The periodontal data included the number of teeth present, O'Leary's plaque control record (PCR) (O'Leary et al. 1972), bleeding on probing (BOP), probing depth (PD), and clinical attachment level (CAL). The reproducibility of periodontal measurements conducted by a calibrated periodontist (T.K.), who was blinded to the comorbidity and rheumatologic data, was confirmed by an intra‐examiner intraclass correlation coefficient of 0.90. The average scores for PCR, BOP, PD, CAL, and the percentages of sites with PD and CAL ≥ 4 mm were calculated for each patient. The periodontal inflamed surface area (PISA) was also determined from BOP and PD measurements for each patient, according to a previously described method (Nesse et al. 2008). The presence of periodontitis was confirmed according to the definitions of the Centers for Disease Control and Prevention and the American Academy of Periodontology (Eke et al. 2012).

### 2.5 Serological Measurements

The serum creatinine levels were measured using an enzymatic method (SRL Co., Hachioji, Japan) (Matsuo et al. 2009). The serum levels of CRP and RF were determined using a simple nephelometric and latex particle‐enhanced method, respectively, and those of anti‐CCP IgG, NETs‐associated myeloperoxidase‐DNA complexes, and PAD‐4 were determined by an enzyme‐linked immunosorbent assay (ELISA), as previously described (Kojima et al. 2016; Shidara et al. 2011; Shimada et al. 2016; Wang et al. 2016). The serum IgG titers against *P. gingivalis* FDC381 sonicated preparations and PPAD were determined by an ELISA using the same method as previously reported (Kudo et al. 2012; Shimada et al. 2016), and those against agalactosyl IgG were evaluated by an electrochemiluminescence immunoassay, as previously reported (Matsui et al. 2006). Positivity for RF, anti‐CCP IgG, and anti‐agalactosyl IgG levels was defined by values of ≥ 15 IU/mL, ≥ 4.5 U/mL, and ≥ 6.0 AU/mL, respectively. Serum NETs and anti‐*P. gingivalis* and anti‐PPAD IgG titers were expressed in ELISA units (EU).

### 2.6 Evaluations of CKD

The presence of CKD was evaluated according to the classification of the Kidney Disease Improving Global Outcomes 2012 clinical practice guideline (Levin and Stevens^2014^), where the severity of CKD was classified by the cause, glomerular filtration rate (GFR), and presence of proteinuria sustained for over 3 months. To assess the GFR, the serum creatinine‐based estimated GFR (eGFR) was calculated using the Japanese eGFR equations (Imai et al. 2009; Matsuo et al. 2009). Proteinuria was defined as a dipstick urinalysis score ≥ 1+ (Imai et al. 2009; Tokoroyama et al. 2017). In the present study, CKD was defined as having either an eGFR < 60 mL/min/1.73 m^2^ or proteinuria, or both. Patients who showed imaging findings, blood abnormalities, or pathological kidney observations were excluded.

### 2.7 Statistical Analyses

The sample size calculation with the SPSS Statistics software program (version 29.0; IBM, Chicago, IL, USA) revealed that including ≥ 8 patients would exceed a statistical power of 0.8 with an alpha level of 5%, which was based on the eGFR in a previous study (Tokoroyama et al. 2017).

After evaluating the normality of distribution using Kolmogorov–Smirnov tests, differences in continuous variables between the groups were assessed using Mann–Whitney *U‐*tests. Chi‐square and Fisher's exact tests were used to compare categorical variables, including the gender (female and male coded as 0 and 1, respectively), smoking status (never‐smoker, former‐smoker, and current‐smoker coded as 0, 1, and 2, respectively), comorbidity (the presence and absence of disease coded as 0 and 1, respectively), and use of RA‐related drugs (non‐use and use as 0 and 1, respectively) between the groups. The significance of the association between the characteristics and eGFR was evaluated by a bivariate analysis with Spearman's rank correlation coefficient as well as by a multiple regression analysis after adjusting for age, gender, smoking status, comorbidity, RA condition, and RA‐related drugs. Furthermore, the significance of the association between the characteristics and CKD (the presence and absence of CKD coded as 0 and 1, respectively) was assessed using a multiple logistic regression analysis after adjusting similarly to above. Statistical significance was set at 5% (*p* < 0.05), except for the multiple regression analysis with Bonferroni correction.

## 3 Results

### 3.1 Comparison of Characteristics Between RA Patients Grouped by CKD

Of the 127 patients, 20 showed an eGFR < 60 mL/min/1.73 m^2^, while none showed proteinuria (Table 1). Therefore, the present study included 20 patients with CKD and 107 patients without CKD, according to the CKD classification (Levin and Stevens^2014^). The CKD group displayed a significantly older age (*p* = 0.002), higher percentages of former smokers (*p* = 0.01), more sites with PD and CAL ≥ 4 mm (*p* = 0.03 and *p* = 0.02), and higher levels of serum creatinine and eGFR (*p* < 0.001 for both) and anti‐*P. gingivalis* IgG titers (*p* = 0.04) than the non‐CKD group (Table 1). However, both groups had similar proportions of use and dosage of glucocorticoids and csDMARDs, including methotrexate, sulfasalazine, bucillamine, tacrolimus, and mizoribine (*p* > 0.05 for all) (Table 2). The frequencies of the use of NSAIDs, TNF inhibitors (i.e. IFX, ETN, ADA, and GLM), and IL‐6RI inhibitors (i.e. TCZ) were also similar between the groups (*p* > 0.05 for all) (Table 2). None of the patients received treatment with iguratimod, CPZ, ABT, or a Janus kinase inhibitor (tofacitinib).

**Table 1:** Characteristics of the study patients with RA and periodontitis.

Characteristics	All patients (*n* = 127)	Non‐CKD group^a^ (*n* = 107)	CKD group^a^ (*n* = 20)	*p* value
*Demographic*				
Age, years	58.1 ± 12.0	56.7 ± 12.2	65.4 ± 7.7	**0.002** *
Female, *n* (%)	104 (81.9)	90 (84.1)	14 (70.0)	0.13
Current‐/former‐/never‐smoker, %	0/20/80	0/16/84	0/40/60	**0.01** *
*Comorbidity*				
Cardiovascular disease	7 (5.5)	5 (4.7)	2 (10.0)	0.34
Hypertension	25 (19.7)	20 (18.7)	5 (25.0)	0.52
Diabetes mellitus	9 (7.1)	7 (6.5)	3 (15.0)	0.20
Dyslipidemia	18 (14.2)	14 (13.1)	4 (20.0)	0.42
*Rheumatologic*				
RA duration, months	88.5 ± 97.2	80.3 ± 86.1	132.1 ± 137.8	0.26
DAS28‐CRP	3.0 ± 1.2	3.0 ± 1.2	2.8 ± 1.0	0.38
Remission/low/moderate/high activity, %	32/16/39/13	32/15/40/13	35/20/30/15	0.71
TJC	2.7 ± 4.3	3.0 ± 4.6	1.7 ± 2.0	0.41
SJC	2.6 ± 3.9	2.7 ± 4.1	1.8 ± 2.8	0.36
Pt‐GA, mm	29.0 ± 24.5	29.8 ± 24.4	25.0 ± 24.9	0.33
*Periodontal*				
The number of teeth present	25.0 ± 3.4	25.0 ± 3.6	25.1 ± 2.6	0.79
PCR, %	41.7 ± 22.0	41.8 ± 22.1	40.9 ± 21.6	0.86
Sites with BOP, %	11.0 ± 15.1	10.3 ± 14.0	14.7 ± 19.8	0.10
PD, mm	2.8 ± 0.4	2.8 ± 0.4	2.8 ± 0.4	0.92
Sites with PD ≥ 4 mm, %	12.1 ± 16.6	11.5 ± 16.7	15.4 ± 15.9	**0.03** *
CAL, mm	2.9 ± 0.5	2.9 ± 0.5	2.9 ± 0.5	0.85
Sites with CAL ≥ 4 mm, %	14.1 ± 18.3	13.2 ± 18.2	19.1 ± 18.5	**0.02** *
PISA, mm^2^	294.2 ± 389.8	285.0 ± 382.0	343.5 ± 436.7	0.80
*Serological*				
Creatinine, mg/dL	0.6 ± 0.2	0.6 ± 0.1	0.9 ± 0.2	< **0.001** *
eGFR, mL/min/1.73m^2^	85.0 ± 23.8	91.1 ± 20.8	52.6 ± 6.0	< **0.001** *
CRP, mg/dL	1.2 ± 2.2	1.4 ± 2.3	0.5 ± 0.8	0.10
RF, IU/mL	124.6 ± 252.5	119.5 ± 256.9	152.1 ± 231.4	0.14
RF positive, *n* (%)	96 (75.6)	78 (72.9)	18 (90.0)	0.10
Anti‐CCP IgG, U/mL	141.2 ± 138.9	140.6 ± 143.6	144.5 ± 114.1	0.55
Anti‐CCP IgG positive, *n* (%)	102 (80.3)	84 (78.5)	18 (90.0)	0.24
Anti‐agalactosyl IgG, AU/mL	169.2 ± 303.4	160.3 ± 300.4	216.5 ± 323.2	0.10
NETs, EU	1.4 ± 0.6	1.4 ± 0.6	1.4 ± 0.5	0.85
PAD‐4, ng/mL	3.1 (1.3)	3.1 (1.3)	3.1 (1.7)	0.82
Anti‐*P. gingivalis* IgG, EU	10.7 ± 13.2	9.4 ± 11.7	18.0 ± 18.0	**0.04** *
Anti‐PPAD IgG, EU	1.3 ± 0.8	1.3 ± 0.8	1.1 ± 0.9	0.15

*Note:* Data are expressed as the mean ± standard deviation, except for the number (%) or %.

Abbreviations: BOP, bleeding on probing; CAL, clinical attachment level; CCP, cyclic citrullinated peptide; CKD, chronic kidney disease; CRP, C‐reactive protein; DAS28, disease activity score in 28 joints; eGFR, estimated glomerular filtration rate; IgG, immunoglobulin G; NETs, neutrophil extracellular traps; PAD, peptidylarginine deiminase; PCR, O'Leary's plaque control record; PD, probing depth; PISA, periodontal inflamed surface area; PPAD, *P. gingivalis* PAD; Pt‐GA, patient global assessment (0‐100 mm); RA, rheumatoid arthritis; RF, rheumatoid factor; SJC, swollen joint count; TJC, tender joint count.

^a^ CKD was defined as an eGFR < 60 mL/min/1.73 m^2^ and/or proteinuria of ≥ 3 months' duration (Levin and Stevens 2014).

* Significantly different between the groups (*p* < 0.05) in bold, as assessed using Mann–Whitney *U‐*test and chi‐square and Fisher's exact tests, respectively.

**Table 2:** RA‐related drugs administered to the study patients with RA and periodontitis.

Drugs	All patients (*n* = 127)	Non‐CKD group^a^ (*n* = 107)	CKD group^a^ (*n* = 20)	*p* value^b^
csDMARDs use, *n* (%)	101 (79.5)	87 (81.3)	14 (70.0)	0.25
Methotrexate use, *n* (%)	76 (59.8)	68 (63.6)	8 (40.0)	0.05
Methotrexate dosage, mg/week	8.5 ± 3.1	8.7 ± 3.1	6.8 ± 2.1	0.12
Sulfasalazine use, *n* (%)	36 (28.3)	31 (29.0)	5 (25.0)	0.72
Sulfasalazine dosage, mg/day	903 ± 201	919 ± 187	800 ± 274	0.22
Bucillamine use, *n* (%)	18 (14.2)	16 (15.0)	2 (10.0)	0.56
Bucillamine dosage, mg/day	183 ± 42	184 ± 44	175 ± 35	0.28
Tacrolimus use, *n* (%)	11 (8.7)	8 (7.5)	3 (15.0)	0.27
Tacrolimus dosage, mg/day	1.8 ± 0.9	1.7 ± 0.9	1.9 ± 1.0	0.67
Mizoribine use, *n* (%)	21 (16.5)	18 (16.8)	3 (15.0)	0.84
Mizoribine dosage, mg/day	321 ± 137	342 ± 135	200 ± 87	0.10
Glucocorticoids use, *n* (%)	78 (61.4)	63 (58.9)	15 (75.0)	0.18
Glucocorticoids dosage, mg/day	4.0 ± 2.5	4.1 ± 2.6	3.4 ± 1.7	0.54
NSAIDs use, *n* (%)	30 (23.6)	26 (24.3)	4 (20.0)	0.68
TNF inhibitor use, *n* (%)	49 (38.6)	44 (41.1)	5 (25.0)	0.18
IFX/ETN/ADA/GLM use, *n*	8/20/16/5	8/17/15/4	0/3/1/1	0.23
TCZ for IL‐6R inhibitor use, *n* (%)	41 (32.3)	36 (33.6)	5 (25.0)	0.45

*Note:* Data are expressed as the mean ± standard deviation, the number (%), or the number.

None of the patients received treatment with iguratimod, certolizumab pegol, abatacept, or tofacitinib.

Abbreviations: ADA, adalimumab; CKD, chronic kidney disease; csDMARDs, conventional synthetic disease‐modifying antirheumatic drugs; eGFR, estimated glomerular filtration rate; ETN, etanercept; GLM, golimumab; IFX, infliximab; IL‐6R, interleukin‐6 receptor; NSAIDs, non‐steroidal anti‐inflammatory drugs; RA, rheumatoid arthritis; TCZ, tocilizumab; TNF, tumor necrosis factor.

^a^ CKD was defined as an eGFR < 60 mL/min/1.73 m^2^ and/or proteinuria of ≥ 3 months' duration (Levin and Stevens 2014).

^b^ Differences in continuous and categorical variables between the groups were assessed using Mann–Whitney *U‐*tests and chi‐square and Fisher's exact tests, respectively.

### 3.2 Association of the Characteristics With the eGFR and CKD

For periodontitis severity‐related characteristics, the percentages of sites with PD and CAL ≥ 4 mm and PISA were used, as these are critical for defining the presence and severity of periodontitis and for quantifying the periodontal inflammatory burden. The percentage of sites with PD ≥ 4 mm, PISA, and anti‐PPAD IgG titers were not associated with the eGFR (*p* > 0.05 for all), whereas the anti‐*P. gingivalis* IgG titers were significantly associated with the eGFR in all patients (*p* < 0.001 for both) in the bivariate and multiple regression analyses (Table 3). The anti‐*P. gingivalis* IgG titers were also significantly associated with CKD in all patients (*p* < 0.001) according to the multiple logistic regression analysis after adjusting for the age, gender, smoking status, comorbidity, RA condition, and RA‐related drugs (Table 4). The percentage of sites with PD ≥ 4 mm, PISA, and anti‐PPAD IgG titers were not associated with CKD (Table 4). It was not possible to subject the percentage of sites with CAL ≥ 4 mm to multivariate analyses due to multicollinearity.

**Table 3:** Significance of association of the periodontitis severity, anti‐*P. gingivalis* IgG, and anti‐PPAD IgG with eGFR using bivariate and multiple regression analyses.

	Bivariate		Multiple regression	
Characteristics	Correlation coefficient	*p* value	Beta (95% CI)	*p* value
Sites with PD ≥ 4 mm	−1.12	0.26	−0.14 (−0.59 to 0.20)	0.32
PISA	−1.06	0.28	−0.21 (−0.03 to 0.01)	0.13
Anti‐*P. gingivalis* IgG	−3.45	< **0.001** *	−0.31 (−0.86 to −0.27)	< **0.001** **
Anti‐PPAD IgG	0.39	0.70	−0.01 (−4.81 to 4.65)	0.97

Abbreviations: 95% CI, 95% confidence interval; Beta, standardized partial regression coefficient; eGFR, estimated glomerular filtration rate; IgG, immunoglobulin G; PD, probing depth; PISA, periodontal inflamed surface area; PPAD, *P. gingivalis* peptidylarginine deiminase; RA, rheumatoid arthritis.

* Significantly associated with eGFR in 127 patients with RA and periodontitis (*p* < 0.05) in bold, as assessed using Spearman's rank correlation coefficient.

** Significantly associated with eGFR in 127 patients with RA and periodontitis (*p* < 0.0038 after Bonferroni correction) in bold, as assessed using a multiple regression analysis after adjusting for the age, gender, smoking status, comorbidity, RA condition, and RA‐related drugs.

**Table 4:** Significance of association of the periodontitis severity, anti‐*P. gingivalis* IgG, and anti‐PPAD IgG with CKD using a multiple logistic regression analysis.

Characteristics	OR	95% CI	*p* value
Sites with PD ≥ 4 mm	1.04	0.94–1.14	0.46
PISA	1.00	0.99–1.00	0.05
Anti‐*P. gingivalis* IgG	1.17	1.07–1.28	< **0.001** *
Anti‐PPAD IgG	0.56	0.17–1.79	0.33

Abbreviations: 95% CI, 95% confidence interval; CKD, chronic kidney disease; IgG, immunoglobulin G; OR, odds ratio; PD, probing depth; PISA, periodontal inflamed surface area; PPAD, *P. gingivalis* peptidylarginine deiminase; RA, rheumatoid arthritis.

* Significantly associated with CKD in 127 patients with RA and periodontitis (*p* < 0.0038 after Bonferroni correction) in bold, as assessed using a multiple logistic regression analysis after adjusting for the age, gender, smoking status, comorbidity, RA condition, and RA‐related drugs.

## 4 Discussion

To our knowledge, this is the first study to demonstrate a significant association between anti‐*P. gingivalis* IgG titers and CKD in patients with RA and periodontitis. Univariate analyses revealed that the patients with CKD were significantly older, had higher percentages of former smokers, and had higher anti‐*P. gingivalis* IgG titers than those without CKD. These observations are consistent with the results of another large‐scale race‐matched cohort study indicating an increased prevalence of CKD with age (Imai et al. 2009; Saisho et al. 2016) and are supported by the evidence that periodontitis, RA, and CKD share risk factors, such as age and smoking (Li et al. 2021; Smolen et al. 2016). Therefore, the older age and higher percentages of former smokers in the CKD patients may be some of the reasons for their worse periodontal condition and higher anti‐*P. gingivalis* IgG titers in comparison to the non‐CKD patients. In addition, the potential causes of CKD in patients with RA and periodontitis are other comorbidities, an RA condition, and the use of RA‐related drugs (Hickson et al. 2014; Karie et al. 2008; Saisho et al. 2016; Tokoroyama et al. 2017). It is therefore necessary to consider these risk factors when performing multivariate analyses. The data showed that the anti‐*P. gingivalis* IgG titers were significantly associated with the eGFR in both bivariate and multivariate analyses, and were also significantly associated with CKD in a multiple logistic regression analysis after adjusting for age, gender, smoking status, comorbidity, RA condition, and RA‐related drugs. These findings are supported by the results of previous studies showing a significant association between serum antibodies to *P. gingivalis* and CKD in non‐RA patients (Iwasaki et al. 2012; Kshirsagar et al. 2007). However, the results indicated a smaller odds ratio for anti‐*P. gingivalis* IgG titers than in other studies, which might be partially due to differences in the patient cohort or the measurement of anti‐*P. gingivalis* IgG titers between other studies (Iwasaki et al. 2012; Kshirsagar et al. 2007) and ours.

**Figure 2:**
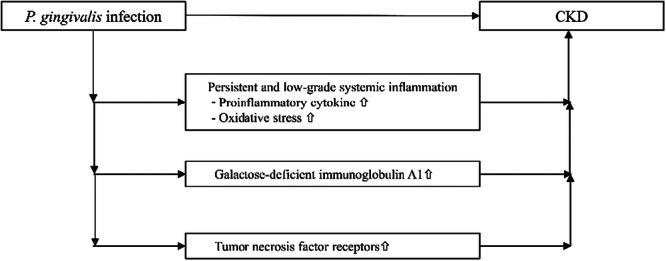
The potential relationship between *Porphyromonas gingivalis* infection and chronic kidney disease (CKD) in patients with rheumatoid arthritis (RA) and periodontitis. ⇧: increased level.

The role of anti‐*P. gingivalis* IgG in CKD pathogenesis remains unclear. Studies have demonstrated that *P. gingivalis* plays a significant role in the progression of RA, including increased protein citrullination through PPAD and *P. gingivalis*‐induced production of pro‐inflammatory cytokines and dysbiosis of the gut microbiota (Bartold and Lopez‐Oliva 2020; Kobayashi and Bartold 2023). However, the data did not show any marked differences in the rheumatologic conditions, as indicated by DAS28‐CRP, TJC, SJC, and Pt‐GA, or in the citrullination‐related characteristics, including anti‐CCP IgG, anti‐PPAD IgG titers, NETs, and PAD‐4, between the patients with and without CKD. These results imply that any association between the anti‐*P. gingivalis* IgG titers and CKD might be due, in part, to persistent and low‐grade systemic inflammation, including increased production of proinflammatory cytokines and oxidative stress, as a result of host responses to oral infection with *P. gingivalis* (Li et al. 2021; Mihai et al. 2018). Alternatively, increased levels of galactose‐deficient IgA1 (Gd‐IgA1) or tumor necrosis factor receptors (TNFR), both of which are induced by *P. gingivalis*, may be involved in patients with RA and periodontitis (Ito et al. 2024; Mikami et al. 2021) (Figure 2). Further studies are required to better understand the association between anti‐*P. gingivalis* IgG titers and CKD by analyzing the serum levels of cytokines, reactive oxygen species, Gd‐IgA1, and TNFR in a larger‐scale patient cohort.

Interestingly, the results failed to show any marked differences in anti‐PPAD IgG levels between the patients with and without CKD, despite their differing levels of anti‐*P. gingivalis* IgG. These findings are in accordance with the results of a previous study that showed differences in serum IgG responses to *P. gingivalis* antigens (Shimada et al. 2016). Therefore, these results suggest that CKD may not be associated with serum IgG response to PPAD in patients with RA and periodontitis.

The present findings for both PD and CAL were comparable, whereas the percentages of sites with PD and CAL ≥ 4 mm were significantly different between the patients with and without CKD. This discrepancy may reflect a greater number of periodontitis‐affected sites in patients with CKD than in those without CKD. Unexpectedly, the present study indicated no association of CKD with the percentages of sites with PD ≥ 4 mm and PISA according to bivariate and multivariate analyses. These findings differ from those of previous studies showing a significant association between CKD and periodontitis in non‐RA patients (Kapellas et al. 2019; Serni et al. 2023). The lack of such an association in the present study might be partly related to the beneficial therapeutic effects of RA‐related drugs, including csDMARDs, corticosteroids, NSAIDs, and bDMARDs, on periodontitis (Han and Reynolds 2012). To confirm and extend these observations, additional studies are needed in a larger number of patients with RA, periodontitis, and CKD, as well as with high exposure to *P. gingivalis*, as measured by increased anti‐*P. gingivalis* IgG titers.

The present study has several methodological limitations that warrant mention. First, retrospective cohort studies such as the present one tend to be associated with selection bias. All study patients were recruited from the Niigata Prefecture and nearby regions. However, the data on the mean age, percentage of females, and prevalence of CKD among patients with RA in the present study were similar to those of another large‐scale race‐matched cohort study (Matsui et al. 2007; Tokoroyama et al. 2017). These observations suggest that the selection bias was minimal. Second, the sample size was relatively small owing to strict eligibility criteria. Patients whose periodontal tissue destruction was mediated by dentures were also excluded. Third, a one‐time assessment of the serum creatinine level may not be an accurate measure of the eGFR because of day‐to‐day variation. Fourth, the revised Japanese eGFR equation, based on serum creatinine levels, was used to assess CKD. However, as the serum creatinine level is affected by muscle volume, it might be more useful to use the creatinine clearance estimated by the Cockcroft‐Gault (CG) equation in elderly Japanese people whose muscle volumes decrease with age (Cockcroft and Gault 1976; Matsuo et al. 2009). Finally, it is desirable to compare the total amount of NSAIDs used between the patients with and without CKD. However, it was difficult to collect accurate patient history data on NSAIDs use from the RA onset.

In conclusion, the results of the present study suggest that serum IgG titers against *P. gingivalis*, but not against PPAD, are associated with CKD in patients with RA and periodontitis. Further studies are required to validate this association using a larger cohort of patients.

## Author Contributions

Tetsuo Kobayashi and Satoshi Ito conceived of and designed the study and wrote the manuscript. Tetsuo Kobayashi made the diagnosis of periodontitis and collected periodontal data. Satoshi Ito, Akira Murasawa, and Hajime Ishikawa were involved in the rheumatologic diagnosis, management, and data acquisition. All authors critically reviewed the manuscript and approved it for submission.

## Ethics Statement

The present study was conducted in accordance with the Declaration of Helsinki, with approval of the Ethics Committee of Niigata University (Approval Number 2017‐0114) and Niigata Rheumatic Center (Approval Number 2017‐009), and the patient signed informed consent.

## Conflicts of Interest

The authors declare no conflicts of interest.

## Data Availability

The data that support the findings of this study are available on request from the corresponding author. The data are not publicly available due to privacy or ethical restrictions. The authors confirm that data supporting the findings of the present study are included within the article, and the remaining data are available from the corresponding author on reasonable request.
